# Unexpected evolutionary diversity in a recently extinct Caribbean mammal radiation

**DOI:** 10.1098/rspb.2014.2371

**Published:** 2015-05-22

**Authors:** Selina Brace, Samuel T. Turvey, Marcelo Weksler, Menno L. P. Hoogland, Ian Barnes

**Affiliations:** 1 School of Biological Sciences, Royal Holloway University of London, Egham, Surrey TW20 0EX, UK; 2 Institute of Zoology, Zoological Society of London, Regent's Park, London NW1 4RY, UK; 3 Laboratório de Ecoepidemiologia da Doença de Chagas, Instituto Oswaldo Cruz, Fiocruz, Avenida Brasil 4365, 21045-900 Rio de Janeiro, Brazil; 4 Departamento de Vertebrados, Museu Nacional, Universidade Federal do Rio de Janeiro, Quinta da Boa Vista, São Cristóvão, 20940-040 Rio de Janeiro, Brazil; 5 Faculty of Archaeology, Leiden University, Einsteinweg 2, 2333CC Leiden, The Netherlands

**Keywords:** ancient DNA, biogeography, extinct mammal, island evolution, Oryzomyini, phylogeny

## Abstract

Identifying general patterns of colonization and radiation in island faunas is often hindered by past human-caused extinctions. The insular Caribbean is one of the only complex oceanic-type island systems colonized by land mammals, but has witnessed the globally highest level of mammalian extinction during the Holocene. Using ancient DNA analysis, we reconstruct the evolutionary history of one of the Caribbean's now-extinct major mammal groups, the insular radiation of oryzomyine rice rats. Despite the significant problems of recovering DNA from prehistoric tropical archaeological material, it was possible to identify two discrete Late Miocene colonizations of the main Lesser Antillean island chain from mainland South America by oryzomyine lineages that were only distantly related. A high level of phylogenetic diversification was observed within oryzomyines across the Lesser Antilles, even between allopatric populations on the same island bank. The timing of oryzomyine colonization is closely similar to the age of several other Caribbean vertebrate taxa, suggesting that geomorphological conditions during the Late Miocene facilitated broadly simultaneous overwater waif dispersal of many South American lineages to the Lesser Antilles. These data provide an important baseline by which to further develop the Caribbean as a unique workshop for studying island evolution.

## 1. Introduction

Island systems and the unusual endemic faunas that often occur on them represent important ‘natural laboratories’ that have played a key part in the development of evolutionary thought since the nineteenth century [1,2]. In particular, the dynamics of island colonization events, the factors that determine the pattern and magnitude of adaptive radiations, and the geography of gene flow in relation to different environmental barriers have all become the subject of extensive research [3–5]. Island species are also particularly vulnerable to human-mediated extinction due to factors such as a lack of native mammalian predators on most oceanic islands, and a substantial proportion of endemic insular biodiversity (especially vertebrate biodiversity) has been lost following human arrival in different island systems [6–9].

Central to our capacity to derive general patterns of colonization, radiation and extinction in island systems is the ability to compare across multiple organismal groups. The insular Caribbean has excellent potential in this context, as it is one of the only oceanic-type island systems to have been colonized by non-volant land mammals. The Caribbean Late Quaternary land mammal fauna was characterized by major evolutionary radiations of endemic lipotyphlan insectivores, megalonychid sloths, platyrrhine primates and several families of often large-bodied rodents [9,10]. Subsequently, the region has witnessed the highest level of mammalian species extinction anywhere in the world during the prehistoric Holocene and post-1500 ad historical era [9–12], and from a Late Quaternary fauna containing more than 100 endemic mammals, only two insectivore species and eight currently recognized capromyid rodent species are probably extant [13,14].

However, reconstructing the evolutionary history of the many island clades that now lack any surviving representatives can be extremely challenging, and this may pose a major barrier to understanding fundamental evolutionary patterns and processes in insular systems. Here, we present the first molecular data from one of the Caribbean region's now-extinct major mammal groups: the insular radiation of oryzomyine rice rats (Cricetidae: Sigmodontinae). These are the only native non-volant mammals known from across most of the Lesser Antilles, and are part of an extremely diverse New World rodent radiation containing 28 extant genera and roughly 130 currently recognized extant species [15–17]. Caribbean oryzomyines are now only represented by highly degraded skeletal material from archaeological or palaeontological sites, or by museum specimens (more than 100 years old) of historically live-caught individuals.

**Figure 1.**
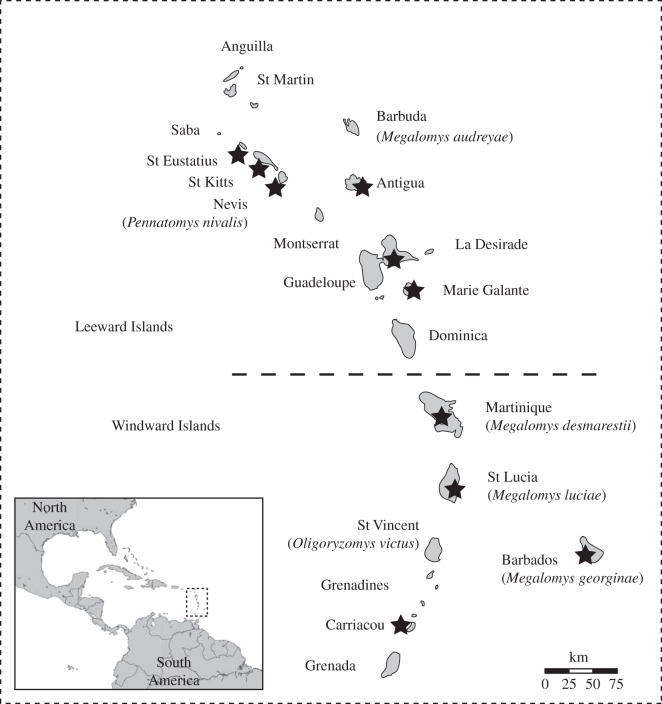
Map showing the distribution of described species of extinct Lesser Antillean oryzomyine rice rats. Starred islands indicate those from which oryzomyine samples used in this study were collected.

Although abundant in the Late Quaternary fossil record and in Holocene pre-Columbian archaeological sites throughout the main Windward–Leeward island chain (figure 1), Caribbean oryzomyine diversity remains unclear. Around 20 separate insular populations, some of which reached the size of small cats [18], have been recorded from different islands between Grenada and the Anegada Passage [19,20]. All of these populations are now extinct, probably as a result of European-era introduction of invasive mammals or agricultural habitat conversion [18]; this dramatic level of extinction is equivalent in magnitude to the much more widely known major historical-era loss of marsupials and rodents in Australia [9,11]. However, only six oryzomyine species (including two endemic genera, *Megalomys* and *Pennatomys*, and a representative of the mainland Neotropical genus *Oligoryzomys* on the southern island of St Vincent) have so far been formally described from the Windward–Leeward island chain [19,20].

Colonization scenarios and intra- and interisland species groupings proposed by previous authors have suggested that oryzomyine taxa from the main Windward–Leeward island chain constitute either a monophyletic clade or separate evolutionary radiations [19,21,22]. Uncertainty also remains over whether oryzomyines reached South America before complete formation of the Isthmus of Panama that facilitated the Great American Biotic Interchange [23,24], and whether their Late Holocene distribution was modified by accidental or deliberate translocation between islands by Amerindians during recent prehistory, a dispersal mechanism which also played an important role in shaping historical distributions of other terrestrial vertebrates in the Lesser Antilles (e.g. iguanas, agoutis, opossums) [25].

Here, we reconstruct oryzomyine colonization of the insular Caribbean and the dynamics of their subsequent evolutionary history. Our specific aims are to determine: (i) whether oryzomyines colonized the main Windward–Leeward island chain north of St Vincent via either a single or multiple waif dispersal events; (ii) levels of phylogenetic diversity, and thus the scale of the subsequent Late Holocene extinction event, shown by oryzomyines across this complex island chain; and (iii) the timing of oryzomyine colonization, diversification and adaptation across the region, particularly in relation to known geological events and prehistoric arrival of Amerindian settlers. This study also has the broader goal of generating new insights into the evolutionary history and Quaternary diversity of the Caribbean mammal fauna, and the wider dynamics of mammalian island colonization events.

## 2. Material and methods

### (a) DNA extraction and amplification

We sampled a total of 42 oryzomyine samples from 10 Lesser Antillean islands (table 1). DNA extractions followed protocols described in [26] and were carried out in a dedicated ancient DNA (aDNA) laboratory at Royal Holloway University of London. Mitochondrial DNA (MtDNA) were amplified in multiple overlapping fragments, averaging 110 base pairs (bp) each, using a total of 18 primer pairs (electronic supplementary material, table S1), targeting a 560 bp region of cytochrome *b* (cyt *b*). All amplifications were repeated (more than two times), sequenced through Sanger and NGS, and sequence data were translated to detect potential NUMTs and miscoding legions. PCR reactions, amplicon purification, Sanger sequencing and protocols to prevent contamination were performed as described in [26].

**Table 1.** Samples of Lesser Antillean extinct oryzomyines investigated for mtDNA analyses. All samples are from pre-Columbian zooarchaeological sites except for two historical (nineteenth-century) soft-tissue samples of *Megalomys desmarestii* (Martinique) and *M. luciae* (St Lucia). BMHS, Barbados Museum and Historical Society; LU, Leiden University; MNHN, Muséum National d'Histoire Naturelle (Paris); NHM, Natural History Museum (London; mammalogy collections); UF, Florida Museum of Natural History, University of Florida (zooarchaeology comparative collections); US, University of Southampton; UW, University of Washington; YPM, Yale Peabody Museum of Natural History.

taxon	island	site	material	no. samples	source
undescribed	Antigua	Indian Creek	bone	5	YPM
*Megalomys georginae*	Barbados	Silver Sands	bone	2	BMHS
undescribed	Carriacou	Sabazan	bone	2	UW
undescribed	Guadeloupe	Anse à la Gourde	bone	5	LU
undescribed	Marie Galante	Taliseronde	bone	5	UF
*Megalomys desmarestii*	Martinique	Paquemar	bone	3	UF
*Megalomys desmarestii*	Martinique	unknown (nineteenth-century wild-caught)	dried tissue	1	NHM
*Pennatomys nivalis*	Nevis	Hichmans	bone	4	US
*Pennatomys nivalis*	St Eustatius	Golden Rocks	bone	7	UF
*Pennatomys nivalis*	St Kitts	Sugar Factory Pier	bone	7	UF
*Megalomys luciae*	St Lucia	unknown (nineteenth-century wild-caught)	dried tissue	1	MNHN

### (b) Library construction and NGS sequencing

For each of the eight samples that successfully amplified mtDNA, the PCR products (four to nine amplicons per sample) were pooled to an equimolar concentration. Libraries for these eight multi-amplicon samples were constructed using a previously described protocol [27] with the following modifications: the initial DNA fragmentation step was not required; all clean-up steps used MinElute PCR purification kits; Buffer Tango and ATP were replaced with 0.1 mg ml BSA and 1 × T4 DNA ligase buffer during the blunt-end repair step; the proceeding clean-up step was replaced by an inactivation step, heating to 75°C for 10 min; 0.5 mM ATP replaced the T4 DNA Ligase buffer during the adapter ligation step. The index PCR step followed a further protocol using *Pfu* Turbo Cx and the addition of 0.4 mg ml BSA [28]. The index PCR was set for 20 cycles with three PCR reactions conducted per library. The eight indexed libraries were diluted to an equimolar concentration and pooled. The multiplexed samples were sequenced on an Illumina MiSeq platform (Natural History Museum, London) using a single lane on a paired-end flow cell.

### (c) Quality control and alignment

Samples were demultiplexed on the MiSeq instrument. *FASTQ* files were trimmed and filtered by quality using the web-based platform Galaxy [29]. PCR primer sequences were removed and SeqPrep (https://github.com/jstjohn/SeqPrep) was employed to remove adapter sequences and merge reads. Reads were aligned to a reference sequence (obtained through our Sanger sequencing) with Bowtie2 [30]. Duplicate sequences were removed using SAMTools [31] and a consensus sequence obtained implementing SAMTools quality scoring in UGene [32].

### (d) Phylogenetic analyses

Ancient DNA sequences were aligned with modern oryzomyine mtDNA cyt *b* sequences available on GenBank (electronic supplementary material, table S2). For extant oryzomyines, we also retrieved and aligned GenBank sequences for two nuclear genes: interphotoreceptor retinoid binding protein (IRBP) and alcohol dehydrogenase (Adh1) intron 2 (electronic supplementary material, table S2). For each gene, DNA substitution model and partition fit were selected under Bayesian information criterion using PartitionFinder 1 [33]. Two partitions were selected for cyt *b*: codon positions 1 and 2 (GTR + G) and codon position 3 (HKY + G). For IRBP, partitioning was rejected and HKY + G was selected for all codon positions. For Adh1, the HKY + G substitution model was selected. For the full dataset (three gene regions), Bayesian trees were constructed using MrBayes v. 3.2 [34], implementing nucleotide substitution models as selected through PartitionFinder, using four chains (three heated, one cold) run for 1 × 10^7^ generations, sampling every 1 × 10^4^ generations with a burn-in period of 2500 trees. Nodal support was determined by approximate posterior probabilities performed in MrBayes. Divergence estimates and mutation rates were independently calculated in BEAST v. 1.7.5 [35] using the cyt *b* dataset with data partitioning and substitution models as selected through PartitionFinder. In all BEAST analyses, the Yule speciation process was implemented. Divergence dating for Caribbean fauna is problematic due to an almost complete absence of any Tertiary mammal fossil record from the insular Caribbean. We therefore applied a strict clock with a mutation rate of 4% per site per million years [36,37] in our divergence date estimates. To directly address the potential significance of known major historical events (3.75 Ma and 7 ka) without applying a fixed mutation rate, our second set of analyses fixed the divergence dates to either 3.75 Ma or 7 ka and estimated the mutation rate required. Two independent runs were conducted for each of the estimated parameters, with chain length set to 1 × 10^7^ generations, data collected every 1 × 10^3^ generations and a burn-in of 1 × 10^5^ generations. Outputs from MrBayes and BEAST runs were examined with Tracer v. 1.5 [38] to check for stabilization and convergence between runs. *Peromyscus truei* was used as the outgroup. For comparison with existing oryzomyine data, pairwise sequence divergence estimates were calculated in MEGA v. 5.1 [39] using the Kimura-2 parameter (K2P) model.

## 3. Results and discussion

### (a) Oryzomyine colonization of the Lesser Antilles: single or multiple events?

To determine the colonization dynamics of the extinct Lesser Antillean oryzomyines, we attempted to sequence a 560 bp region of mtDNA cyt *b* from a total of 42 ancient specimens collected from 10 islands (figure 1 and table 1). Eight specimens from seven islands (Antigua, Guadeloupe, Martinique, Nevis, St Eustatius, St Kitts and St Lucia) successfully yielded mtDNA. This low success rate was an expected outcome due to the known low preservation potential of ancient biomolecules from tropical island systems, which also prohibited attempts to amplify nuclear DNA.

**Figure 2.**
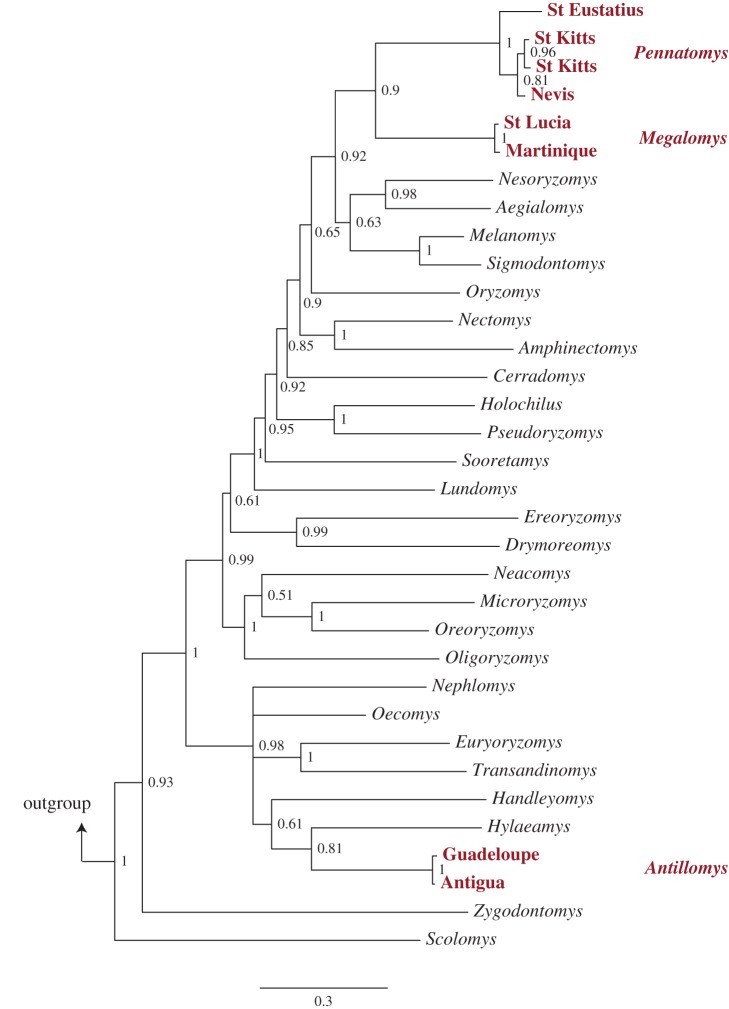
Oryzomyine phylogeny inferred from mtDNA (cyt *b*) and nuclear (IRBP and Adh1) sequence data, showing Bayesian posterior probabilities of nodes. Extinct Lesser Antillean oryzomyine taxa are highlighted in bold; outgroup (*Peromyscus truei*) removed for display purposes. Scale bar represents number of substitutions per site. (Online version in colour.)

Bayesian phylogenetic analyses of our aDNA data combined with modern data from 26 extant sigmodontine genera from continental Central and South America and the Galápagos Islands (electronic supplementary material, table S2) clearly identified two distinct colonization events of the main Windward–Leeward island chain north of St Vincent (figure 2). The endemic Antillean taxa *Megalomys* (Martinique, St Lucia) and *Pennatomys* (Nevis, St Eustatius, St Kitts) are demonstrated to be sister genera, in contrast to the conclusions of previous morphology-only phylogenetic analyses [19,20]. The monophyletic *Megalomys*–*Pennatomys* clade is distributed widely across the Lesser Antilles, and is most closely related to a clade containing representatives from both the mainland Neotropics (Trans-Andean South America and Central America; *Aegialomys*, *Melanomys*, *Sigmodontomys*) and the Galápagos Islands (*Aegialomys*, *Nesoryzomys*). These taxa together form part of a wider oryzomyine clade (‘Clade D’ of [15]), which has undergone extensive radiation throughout the oceanic and continental shelf islands of the Neotropical region (also including Curaçao, Fernando de Noronha, Jamaica and Trinidad [15,19]), and which contains several taxa that possess semi-aquatic adaptations (e.g. natatory fringes, interdigital membranes) and are associated with marshes, rivers, streams or coastlines, probably predisposing them to accidental overwater waif dispersal [19].

By contrast, oryzomyines from Antigua and Guadeloupe, representing taxonomically undescribed populations, together constitute a separate monophyletic clade not predicted by previous published studies, occurring on islands situated geographically intermediate between the distributions of *Megalomys* and *Pennatomys* along the Windward–Leeward island chain. This clade appears most closely related to the mainland South American genus *Hylaeamys*, and falls within a different wider oryzomyine clade (‘Clade B’ of [15]). Extant members of this clade are primarily inhabitants of forest environments in Central and South America, occurring across the Amazonian and Orinoco basins [40]. Although not having the same suite of semi-aquatic adaptations as members of Clade D, *Hylaeamys* and closely related genera occur along riverbanks and so could become associated with natural rafts of floating vegetation for overwater dispersal. The high posterior probability value for the placement of the Antigua–Guadeloupe clade (0.98) and even stronger support for the deep genetic divergence that separates the Antigua–Guadeloupe and *Megalomys*–*Pennatomys* clades (1.00) demonstrate distinct evolutionary histories for these taxa, revealing that the main Windward–Leeward island chain north of St Vincent was colonized separately from South America by at least two oryzomyine lineages that were only distantly related.

### (b) Antillean oryzomyine diversity

A second unexpected result of our aDNA analyses was the high level of phylogenetic diversification observed within the recently extinct oryzomyine radiation across the Lesser Antilles. Although overall endemism is high within the main Windward–Leeward island chain, Antillean terrestrial vertebrate taxa are typically distributed across all major islands situated together on shallow submerged banks, which would have been exposed above water during repeated Quaternary low sea-level stands, thus periodically removing barriers to gene flow between neighbouring populations. Many Antillean vertebrates are therefore interpreted as ‘bank endemics’, with little differentiation usually observed between allopatric populations on the same island bank [41]. In particular, the St Kitts Bank (comprising Nevis, St Eustatius and St Kitts) displays a particularly low level of bank endemism in its reptile fauna compared with other Lesser Antillean island groups, suggesting greater potential for dispersal of terrestrial vertebrate populations across this part of the Windward–Leeward island chain [41].

Oryzomyines from Nevis, St Eustatius and St Kitts were originally interpreted as conspecific and all assigned to *Pennatomys nivalis*, as fragmentary craniodental material available from Holocene zooarchaeological deposits on the three islands showed minimal apparent morphological differentiation [19]. However, our analyses reveal that oryzomyines from these islands display considerable genetic differentiation from each other, suggesting that gene flow between neighbouring island populations on the St Kitts Bank was surprisingly limited throughout the Quaternary. Interisland cyt *b* sequence divergence in oryzomyine populations on the St Kitts Bank ranges from 2.16% (Nevis–St Kitts) to 7.16% (Nevis–St Eustatius; table 2). This level of sequence divergence is comparable with the range reported between morphologically distinct species in some living sigmodontine rodent genera (e.g. *Oecomys*: 1.23–21% [42]), although similar levels of sequence divergence are also observed between allopatric populations of other sigmodontine species (e.g. *Hylaeamys megacephalus*: 0.13–9.13% [43]). While the taxonomic status of now-extinct oryzomyine populations on the St Kitts Bank therefore remains unresolved in the absence of further genetic or morphological data, these island populations had clearly experienced significant diversification and represent a relatively substantial, unexpected within-bank evolutionary radiation.

**Table 2.** Pairwise estimates of cytochrome *b* sequence divergence (%) between oryzomyine samples from different Lesser Antillean islands.

	St Kitts a	St Kitts b	St Eustatius	Nevis	St Lucia	Martinique	Antigua
St Kitts a							
St Kitts b	0.85						
St Eustatius	5.74	6.68					
Nevis	2.16	2.16	7.16				
St Lucia	11.1	10.1	12.7	10.6			
Martinique	11.1	10.1	12.7	10.6	0		
Antigua	16.2	15.1	15.8	16.7	11	11	
Guadeloupe	16.2	15.1	15.8	16.7	11	11	0

Estimates of cyt *b* sequence divergence between oryzomyines on different island banks show even greater differentiation (table 2), supporting morphology-based genus-level differentiation between *Megalomys* and *Pennatomys* [19], and also supporting the distinctiveness of the Antigua–Guadeloupe lineage both from other Antillean oryzomyine genera (minimum sequence divergence = 11.0%, from *Megalomys* on Martinique–St Lucia) and from its mainland sister genus *Hylaeamys* (sequence divergence = 14.7%). These high sequence divergence estimates, and phylogenetic placement of the Antigua–Guadeloupe lineage far from the monophyletic *Megalomys*–*Pennatomys* clade within the wider oryzomyine radiation, both support our independent morphological assessment of the distinctiveness of the Antigua–Guadeloupe lineage. We therefore recognize it as being distinct at the genus level from other oryzomyines, and describe it here as the new genus and species *Antillomys rayi* (electronic supplementary material, appendix, systematic paleontology and figure S1).

### (c) Temporal diversification

In order to examine patterns of temporal diversification in the two Lesser Antillean oryzomyine clades, we employed molecular clock analyses and a median (4% per site per million years) estimate for rodent cyt *b* mutation rates [36,37] (table 3; electronic supplementary material, appendix and figure S2). Both *Antillomys* and the *Megalomys*–*Pennatomys* clade are estimated to have diverged from the most recent common ancestor that each lineage shared with mainland South American oryzomyine sister taxa at very similar times, probably both during the Messinian Stage of the Late Miocene; *Antillomys* is estimated to have diverged at 6.303 Ma (95% HPD, 4.243–8.442 Ma), whereas the *Megalomys*–*Pennatomys* clade is estimated to have diverged at 6.814 Ma (95% HPD, 5.259–8.474 Ma). *Megalomys* and *Pennatomys* are also likely to have diverged from each other during the Messinian, with an estimated divergence date of 5.471 Ma (95% HPD, 3.823–7.288 Ma). Infrageneric diversification in *Antillomys*, *Megalomys* and *Pennatomys* is estimated to have occurred considerably later, during the Middle–Late Pleistocene: *Antillomys* = 0.097 Ma (95% HPD, 0.001–0.235 Ma), *Megalomys* = 0.127 Ma (95% HPD, 0.009–0.276 Ma) and *Pennatomys* = 1.209 Ma (95% HPD, 0.584–1.907 Ma). Notably, diversification within *Pennatomys* pre-dates the divergence of *Megalomys* populations on Martinique and St Lucia; as these *Megalomys* populations are recognized as morphologically distinct species (*M. desmarestii* and *M. luciae*) that vary in body mass as well as both craniodental and soft-tissue characteristics [18–20], this provides further support for likely species-level differentiation between the morphologically more poorly understood *Pennatomys* populations on the St Kitts Bank. Conversely, diversification within *Antillomys* is very recent and post-dates divergence of other Antillean oryzomyine lineages, consistent with a lack of morphological differentiation between *Antillomys* populations on different islands (electronic supplementary material, appendix, systematic paleontology).

**Table 3.** Date estimates (Ma) for the most recent common ancestor between selected oryzomyine taxa based on a moderate estimate (4% per site per million years) for rodent cyt *b* mutation rates (cyt *b*-only data).

taxa	divergence date estimates		
	mean date	95% HPD lower	95% HPD upper
St Kitts and Nevis	0.343	0.135	0.586
St Kitts, Nevis and St Eustatius	1.209	0.584	1.907
St Lucia and Martinique	0.127	0.009	0.276
St Lucia, Martinique, St Kitts, Nevis and St Eustatius	5.471	3.823	7.288
St Lucia, Martinique, St Kitts, Nevis, St Eustatius, *Nesoryzomys* and *Aegialomys*	6.814	5.259	8.474
Antigua and Guadeloupe	0.097	0.001	0.235
Antigua, Guadeloupe and *Hylaeamys*	6.303	4.243	8.442

Additional analyses were used to explore the potential significance of known major historical events in driving the evolution and diversification of the Antillean oryzomyine fauna. The hypothesis that colonization of the insular Caribbean from mainland South America by oryzomyines was not possible before the closing of the Isthmus of Panama and establishment of a continuous terrestrial colonization route between North and South America around 3.5–4 Ma [44] was investigated by fixing the divergence date between island oryzomyine taxa and their mainland South American sister taxa to 3.75 Ma (s.d. = 0.5 Myr). The resultant mutation rate estimates generated under this model (electronic supplementary material, appendix, table S3), while faster than our initial moderate (4% per site per million years) mutation rate estimate, are not unfeasible. However, our models suggest that substantial diversification within the wider Neotropical oryzomyine clade probably occurred prior to the closing of the Isthmus, lending support to previous suggestions that oryzomyines first reached South America via transient island chains before the main phase of the Great American Biotic Interchange [23,24]. As some other terrestrial Caribbean vertebrates (e.g. *Spondylurus* skinks) may have speciated as recently as the mid-Holocene in response to post-glacial geographical isolation [45], further analysis was also conducted to investigate the possibility that interisland oryzomyine diversification may have been driven by supposed prehistoric Holocene Amerindian translocation [25]. However, even when the most recent oryzomyine inter-island divergence dates were fixed to the oldest suggested date for Amerindian occupation of the Lesser Antilles (approx. 7 ka, s.d. = 0.1 kyr [10,25]), the estimated mean mutation rates required (87–212%) are absurdly fast (electronic supplementary material, appendix, table S3).

### (d) Comparative dynamics of island colonization and evolution

The unexpected and complex evolutionary history of the recently extinct Lesser Antillean oryzomyine radiation revealed by our aDNA analyses has wider implications for understanding the biological history of the insular Caribbean and other island systems. Different non-volant vertebrate lineages, including multiple mammal lineages, appear to have reached the Greater Antilles at various times throughout the Neogene, Palaeogene and Mesozoic via a complex series of overwater dispersals, transient land bridges and vicariance events [2,10,45–51]. By contrast, although the Lesser Antillean volcanic arcs have been active since the Eocene [46,52], the region's modern non-volant vertebrate fauna is considerably younger in age. Whereas some non-volant vertebrate taxa (e.g. eleutherodactyline frogs [50], West Indian racers [51]) reached the main Lesser Antillean island chain from the Greater Antilles, other groups colonized directly from mainland South America, presumably through similar dispersal events to those that probably mediated the arrival of oryzomyines. Interestingly, other recent molecular studies indicate that numerous different amphibian and reptile taxa (frogs *Allobates chalcopis* and *Leptodactylus fallax*; pit-vipers *Bothrops* spp.; mabuyine skinks *Capitellum* spp., *Mabuya* spp. and *Spondylurus* spp.) reached the Lesser Antilles from South America during the Late Miocene, sharing a closely similar age to both the *Antillomys* and the *Megalomys–Pennatomys* lineages [45,53]. The Late Miocene was an interval of low eustatic sea level during which a northward riverine connection between Amazonia and the Caribbean may have existed [54], therefore potentially providing favourable geomorphological conditions that facilitated broadly simultaneous overwater waif dispersal of many South American mainland vertebrate lineages to the Lesser Antilles.

Infrageneric diversification in each of the Lesser Antillean oryzomyine genera took place during the Middle–Late Pleistocene, an interval when glacial cycles were driving major changes in sea level, island area and climate, and therefore facilitating isolation and speciation in many Caribbean island lineages [45,51]. However, oryzomyine populations from islands on the shallow St Kitts Bank, which were regularly reconnected during glacial cycles, retained an unexpectedly high degree of genetic structuring throughout the Pleistocene despite the potential for periodic gene flow between neighbouring populations. Comparable patterns of substantial phylogenetic differentiation maintained across periodic seaways are considered uncommon among insular vertebrates. However, other recent molecular studies have also demonstrated evidence for within-bank differentiation in groups such as West Indian racers (*Alsophis*) on some Lesser Antillean islands [51], as well as in some other mammal taxa occurring on continental shelf islands that have been regularly reconnected to neighbouring mainland populations of sister taxa (e.g. Hainan gibbon *Nomascus hainanus* [55]), suggesting that such insular phylogeographic structuring may be more common than previously supposed. The increasing use of molecular approaches for reconstructing phylogenetic relationships between different Caribbean vertebrate populations is likely to yield further substantial insights into the evolutionary dynamics of island lineages, and we encourage new investigation of other recently extinct Caribbean mammal lineages using aDNA techniques. We also highlight the unexpected diversity of the Lesser Antillean oryzomyine radiation, which is not predicted by previous morphology- or biogeography-based hypotheses of regional mammal evolution, and which emphasizes the severity of the recent Caribbean mammal extinction event.

## Supplementary Material

Systematic Paleontology

## Supplementary Material



## Supplementary Material

SI_Fig_2
