# Supplementary material for: Unexpected evolutionary diversity in a recently extinct Caribbean mammal radiation
Source: Proc Biol Sci. 2015 May 22;282(1807):20142371. doi: 10.1098/rspb.2014.2371 (PMC4424637; doi:10.1098/rspb.2014.2371)
Supplement: Systematic Paleontology [file rspb20142371supp1.docx]

**Supplementary Information**

**Systematic paleontology**

Rodentia Bowditch 1821

Muroidea Illiger 1811

Cricetidae Fischer 1817

Sigmodontinae Wagner 1843

Oryzomyini Vorontzov, 1959

**Genus *Antillomys* gen. nov.**

**Type species:** *Antillomys rayi* sp. nov.

**Etymology:** After the Antilles.

**Diagnosis:** Differs from other Antillean oryzomyines in the following combination of characters: nasal bones with blunt posterior margins, extending posteriorly approximately at same level as lacrimal bones; lacrimals with maxillary and frontal sutures of similar lengths; interorbital region symmetrically constricted, frontal with squared (angular) relief of dorsal and lateral facies and without supraorbital ridges; incisive foramina very small, not extending posteriorly between M1 alveoli, teardrop-shaped; palate with one small posterolateral palatal pit at each side of mesopterygoid fossa; mesopterygoid fossa extending anteriorly between molar rows; M1 anterocone divided by anteromedian flexus; M2 protoflexus absent; anterolophid absent on m2-3; M1 without accessory labial root (four roots total); m1-3 with two roots.

***Antillomys rayi* gen. et sp. nov.**

**Holotype:** Partial skull, Florida Museum of Natural History (University of Florida), zooarchaeology comparative collections A.98.2 (Figure S1a-c).

**Type locality:** Indian Creek (ad 900-1100 archaeological site), Antigua (see Jones 1985 and Rouse & Morse 1999 for dates and further details for this site).

**Distribution:** Antigua, Barbuda, Guadeloupe and Marie Galante.

**Other examined material:** Named specimen repositories: NHM, Natural History Museum (London), paleontology collections; UF, Florida Museum of Natural History (University of Florida), zooarchaeology comparative collections; G, Musée Edgar Clerk, Le Moule, Guadeloupe. **Antigua**, Indian Creek: UF Zooarch. A36 (maxillary), UF Zooarch. A47 (maxillary), UF Zooarch. A98 series (44 dentaries, maxillaries and premaxillaries), UF Zooarch. A.98.5 (dentary); **Barbuda**, Pleistocene Cave: NHM Paleo. M26901 (several dentaries and maxillaries); Cave II, Two Feet Bay: NHM Paleo. M20210 (dentary); UF Zooarch., uncatalogued skull; **Guadeloupe**, G-30 (dentary), G-34 (maxillary), G-35 (maxillary), G-16 (dentary), G-01 (dentary), G-series (31 dentaries and maxillaries), G-36 (maxillary); **Marie Galante**, Taliseronde, Pits 1 and 2: UF Zooarch. series (several dentaries, maxillaries and humeri) (Figure S1d-i). Other than several specimens from Barbuda, all of this material is late Holocene (pre-Columbian, >500 ya) in age.

**Etymology:** After Clayton Ray, paleontologist who first identified the distinctiveness of *Antillomys*.

**Diagnosis:** Differs from other sigmodontine rodents in the following combination of features: very large size, as large or larger than any extant sigmodontine; stout and wide rostrum; dual articulation of lacrimal with maxillary and frontal; symmetrically constricted anterior interorbital region without supraorbital crests; incisive foramen very short and teardrop-shaped; short bony palate (mesopterygoid fossa extends anteriorly between M3); capsular process present in mandibular ramus; M1 with divided anterocone, well developed mesoloph, and anterior protocone-paracone crista; M2 without protoflexus, and mesoflexus with single internal fossette; M3 with developed mesoloph, small posteroloph, and hypoflexus persistent after moderate wear; m1 with enclosed anteromedian fossettids but lacking anteromedian flexid, ectolophid or ectostylid; mesolophid and mesostylid present, connected to entoconid by lingual cingulum; M1-3 with anterolabial cingula; M1 with four roots; M2-M3 with three roots; m1-3 with two roots.

**Description:** Skull large and robust, with stout and wide rostrum flanked by deep zygomatic notches; interorbital region symmetrically constricted (hourglass shaped), without supraorbital ridges; braincase squared, with very subtle temporal crests. Nasal bones with blunt posterior margins, extending posteriorly approximately at the same level as the lacrimal bones; premaxillaries extending at about same level as nasal; lacrimals with maxillary and frontal sutures of similar lengths. Interorbital region hourglass shaped, frontal with squared (angular) relief of dorsal and lateral facies, without supraorbital ridges. Parietals with broad lateral expansions, a large portion dipping below the temporal ridge posteriorly. The zygomatic plate lacks an anterodorsal spinous process, and its posterior margin lies level to the alveolus of M1. Incisive foramina very small, not extending posteriorly between M1 alveoli, teardrop-shaped. The palatal bridge lacks deep furrows or median ridges, and the bony palate is small, with the mesopterygoid fossa extending anteriorly between molar rows; palate with one small posterolateral palatal pit at each side of mesopterygoid fossa. The posterior portion of all preserved skulls is broken, and thus most information regarding the basicranium is not available. Mental foramen situated at lateral surface of mandible body; capsular process of lower incisor present, ranging from reduced to well developed (polymorphic). Masseteric ridges can form a single open chevron or be conjoined anteriorly (polymorphic); anterior edge of ridges ventral to m1. Incisors ungrooved and without anterolateral bevel. Molars bunodont; M1 without accessory labial root (four roots total), M2 and M3 with three roots each; lower molars with two roots each. Labial cingula closing labial flexi present; incipient lophodonty, flexi of opposite sides interpenetrate planes. M1 anterocone well developed (equal in length and width to protocone-paracone), and divided by anteromedian flexus. Anteroloph reaching labial margin, separated from anterocone by short anteroflexus, which can disappear with slight wear. Protostyle absent; protoflexus broad and deep, with large, gently squared apex. Paraflexus transversely oriented from labial wall, deflected posteriorly close to crown midline and extended along entire length of paracone. Mesoloph well developed; mesoflexus long, transverse, reaching midline of tooth. Paracone connected by enamel bridge to anterior moiety of protocone (preprotocrista); median mure (prehypocrista) connected to posterior moiety of protocone (postprotocrista). Hypoflexus slightly deeper than protoflexus. Metaflexus deep, crescentic, extending over 50% distance across crown and almost reaching hypoflexus. Posteroflexus small, transverse notch at posterior margin of metacone. Posteroloph discernible on worn teeth. M2 protoflexus absent; a small indention anterior to protocone might be present. Mesoflexus present as single internal fossette; paracone without accessory loph. Paraflexus slightly posterolinguad, extending 50% distance across crown. Hypoflexus very deep, sometimes with slightly rounded, expanded apex, and anteroposteriorly shorter than on M1. Metaflexus crescentic, deep and broad, extending well over 50% distance across crown. Posteroflexus very small and faint, apparently apically bifurcated. M3 with developed mesoloph and small posteroloph (discernible from metacone by internal fossette). Hypoflexus present, small but persistent after moderate wear. Paraflexus broad and deep on unworn teeth, becoming greatly reduced by wear; can form separate small internal fold adjacent to apex. Mesoflexus large, transverse; can become isolated as an island. Paracone transverse, anteroposteriorly short or triangular; almost isolated by paraflexus and mesoflexus. Anteroconid well developed, connected to protoconid by paracristid; anteromedian flexid of m1 absent or vestigial, but large anteromedian fossettid apparent in unworn teeth; anterolabial cingulum of m1 present; ectolophid and ectostylid absent; mesolophid present, well developed on m1 and m2 but sometimes joined to entoconid. Anterolabial cingulum present but anterolophid absent on m2 and m3. Posteroflexid of m3 present, well developed. Holotype measurements: length of molar series (occlusal) = 9.32 mm; length of incisive foramina = 5.13 mm; length of diastema = 13.70 mm; breadth of zygomatic plate = 6.53 mm; minimum interorbital width = 7.44 mm.

**Remarks:** Three characters are variable within the sampled material of *Antillomys*: size of capsular process of the lower incisor alveolus; shape of anterior connection of the masseteric ridges; and presence of a supratrochlear foramen in the humerus. Although examined material of *A*. *rayi* displays some morphological variation, no consistent morphological differences are observed between *Antillomys* populations on the Antigua–Barbuda or Guadeloupe banks, and our assignment of *Antillomys* material from Guadeloupe and Marie Galante to *A*. *rayi* is based on the close morphological similarity shown to material from Antigua and Barbuda.

Oryzomyine material from Barbuda was referred to as “*Ekbletomys hypenemus*” by Ray (1962), but this name is not available as it was only reported in an unpublished PhD thesis. The only oryzomyine taxon formally described from within the geographic range of *A*. *rayi* is “*Megalomys*” *audreyae*, known only from a poorly preserved dentary and incisor from “Pleistocene cave-breccia” (specific locality and stratigraphic context unknown) on Barbuda (Hopwood 1925; see also Turvey *et al*. 2012 for further details). Although this taxon is based on very limited material, it displays several morphological and morphometric characteristics that distinguish it from *A*. *rayi*. While *A. rayi* specimens always show a capsular process of the lower incisor alveolus, the only available dentary of *M. audreyae* (NHM Paleo. M7406) does not show any evidence of this process. In addition, the available *M. audreyae* dentary possesses an alveolus for an additional rootlet in the lingual position of m1, whereas no *A. rayi* specimens have such an additional rootlet. The alveolar length of the mandibular toothrow of *M. audreyae* (8.30 mm) is much smaller than that shown by any specimens of *A. rayi* (9.24−10.32 mm, mean = 9.72 mm; n = 40, including specimens from Antigua, Guadeloupe and Barbuda); this difference is statistically significant in a one-sample t-test (t = 29.8, p<0.001). Additional paleontological research on Barbuda is necessary to further evaluate the phylogenetic status of *M. audreyae*, and the stratigraphic relationship between material assigned to *M. audreyae* and *A. rayi*.

*Antillomys* differs from its sister taxon *Hylaeamys* (Figure 2) in several cranial and dental characters: the interorbital region of *Hylaeamys* is slightly anteriorly convergent with weakly developed supraorbital ridges, while in *Antillomys* the interorbital region is hourglass-shaped without any raised ridge or beads; in *Hylaeamys* the parietals are restricted to the dorsal surface of the braincase, while in *Antillomys* the parietals are expanded onto the lateral surface of the braincase; the mesopterygoid fossa of *Hylaeamys* does not extend anteriorly between the maxillary bones, while in *Antillomys* the mesopterygoid extends between the molar tooth rows; the posterolateral palatal pits in *Hylaeamys* are conspicuous large perforations, while in *Antillomys* the pits are small foramina; and the capsular process is absent in *Hylaeamys*, but present in *Antillomys*. Dentally, the anterocone of M1 is undivided in *Hylaeamys* and divided into labial and lingual conules by an anteromedian flexus in *Antillomys*; the paracone is connected to the protocone by an posterior enamel bridge in *Hylaeamys*, but by an anterior bridge in *Antillomys*; a protoflexus is present on M2 and a posteroloph is present on M3 in specimens of *Hylaeamys*, but consistently absent in *Antillomys*; and ectolophids and ectostylids are present in *Hylaeamys* but not in *Antillomys*.

**References**

Hopwood AT (1926) A fossil rice-rat from the Pleistocene of Barbuda. *Ann Mag Nat Hist Ser* 9(17):328-330.

Jones AR (1985) Dietary change and human population at Indian Creek, Antigua. *American Antiquity* 50:518-536.

Ray CE (1962) *Oryzomyine rodents of the Antillean subregion*. Unpublished PhD thesis, Harvard University.

Rouse I, Morse BF (1999) *Excavations at the Indian Creek site, Antigua, West Indies.* Yale University Publications in Anthropology 82, Peabody Museum of Natural History, New Haven, CT.

Turvey ST, Brace S, Weksler M (2012) A new species of recently extinct rice rat (*Megalomys*) from Barbados. *Mamm Biol* 77:404-413.

**Figure S1.** *Antillomys rayi* craniodental material. a-c, partial skull (holotype, UF A.98.2): a, dorsal view; b, ventral view; c, lateral view. d, left premaxilla (UF A.98 series), lateral view. e, h, left dentary (UF A.98.20): e, internal view; h, external view. f, left maxillary (UF A.98 series), occlusal view. g, i, left dentary (UF A.98.23): g, occlusal view; i, external view. Scale bar = 5 mm.

**Figure S2.** Oryzomyine phylogeny (from Figure 2) including date estimates (Mya) for the most recent common ancestor between selected oryzomyine taxa. Arrows point to the dated nodes. An approximate geological timeline (Mya) is illustrated below the phylogeny.**Table S1.** Details of the primer pairs used in our mtDNA (cytochrome *b*) analyses.

| Forward Sequence 5’ to 3’ | Reverse Sequence 5’ to 3’ | Annealing Temp (^0^C) |
| --- | --- | --- |
| ATTTATACTCAACGAAACCTGAAA | CGATGTATGGGATTGCTGA | 50 |
| TAACTACGGCTGACTAATCCGATA | GTTGTGAGTAATAGGATGATTCCAAT | 51 |
| CCGACACAGCTACAGCATT | TGAAGGATCCGTAGTAAATACCTC | 52 |
| ACCATGAGGCCAAATATCATTCTGAG | GGATGAAGTGGAAGGCGAAAAATC | 54 |
| TTTGAGGGGGCTTCTCAGT | AGTAAGGGTGGAATGGGATTTT | 53 |
| CCAATGGAGCCTCAATATTCTT | TAGCCTACGAATGCTGTTGC | 50 |
| CTAAAAGAAACCTGAAACATTGG | GGATAGCTGATAGGAGGTTTGT | 50 |
| CATGCTAATGGAGCTTCCATATT | GCCTACGAATGCTGTTGCTAT | 50 |
| TGAAACCTGAAACATCGGAAT | CCAATGTAGGGAATTGCTGA | 52 |
| TCCCATGAGGCCAAATATC | GGATAAAGTGGAAGGCAAAGA | 52 |
| ATTCTTCATCTGCCTTTTCA | CCTCAGAATGATATTTGTCCTC | 50 |
| GGCTCCAATAACCCCTCAGG | CAGGTGTATAATTATCGGGGTCTCC | 52 |
| CGGAACTACACTAGTAGAATGAAT | TTGTTTGATCCTGTTTCGT | 52 |
| TCCTTCATGCTCACTGAAA | GGCTGATAGGAGGTTTGTAAT | 50 |
| GCCAATGGAGCCTCAATA | GCCTACAAATGCTGTTGCT | 50 |
| CCTGAAACATTGGAATCAT | GTAGTTCCGATGTAAGGGA | 49 |
| CCGACACAGCTACAGCATT | GCGGCCGATATGTATAAATAAA | 52 |
| TACCATGAGGCCAAATATCA | GAAGTGGAAGGCGAAGAAT | 52 |

**Table S2.** Details of the oryzomyini sequences retrieved from GenBank and the oryzomyini sequenced in this study.

| **Species** | **Accession Number Cyt *b*** | **Accession Number IRBP** | **Accession Number Adh1** |
| --- | --- | --- | --- |
| *Hylaemys laticeps* | EU579498 | EU649050 | EU648991 |
| *Transandinomys bolivaris* | EU579513 | EU649073 | EU649030 |
| *Euryoryzomys nitidus* | EU579485 | EU649041 | EU648981 |
| *Oecomys catherinae* | EU579507 | AY163605 | EU649009 |
| *Handleyomys rostratus* | EU579492 | EU649046 | EU648987 |
| *Nephlomys albigularis* | EU579505 | AY163614 | EU649006 |
| *Neacomys spinosus* | EU579504 | KC953406 | EU649002 |
| *Oligoryzomys fulvescens* | GU393997 | EU649063 | EU649014 |
| *Micromys minutus* | EU258535 | AY163592 | EU648999 |
| *Oreoryzomys balneator* | EU579510 | AY163617 | EU649016 |
| *Aegialomys xanthaeolus* | EU074632 | EU273420 | EU648976 |
| *Melanomys caliginosus* | EU340020 | EU649052 | EU648995 |
| *Sigmodontomys alfari* | EU074635 | AY163641 | EU649027 |
| *Oryzomys palustris* | EU074640 | EU273433 | GQ178279 |
| *Nectomys squamipes* | EU074634 | EU273419 | EU649004 |
| *Holochilus chacarius* | GU185898 | EU649048 | DQ227456 |
| *Sooretamys angouya* | EU579512 | EU649072 | EU649029 |
| *Cerradomys scotti* | EU579482 | EU649040 | EU648978 |
| *Pseudoryzomys simplex* | EU579516 | EU649070 | EU649024 |
| *Ereoryzomys polius* | EU579483 | AY163624 | EU648980 |
| *Zygodontomys cherriei* | EU579520 | AY163646 | EU648971 |
| *Drymoreomys albimaculatus* | GU126516 | GU126515 | EU648982 |
| *Amphinectomys savamis* | EU579480 | AY163579 | EU648977 |
| *Lundomys molitor* | JQ966236 | AY163589 | EU648994 |
| *Nesoryzomys fernandinae* | EU579506 | EU649058 | EU649007 |
| *Scolomys ucayalensis* | EU579518 | AY163638 | EU649025 |
| **Species** | **Accession Number Cyt *b*** | **Accession Number IRBP** | **Accession Number Adh1** |
| *Peromyscus truei* | AF108703 | AY277413 | FJ214677 |
| *Antillomys rayi* (Antigua) | LN810048 (561 bp) |  |  |
| *Pennatomys nivalis* (Saint Eustatius) | LN810049 (326 bp) |  |  |
| *Antillomys rayi* (Guadeloupe) | LN810050 (561 bp) |  |  |
| *Pennatomys nivalis* (Saint Kitts) | LN810051 (412 bp) |  |  |
| *Megalomys luciae* (Saint Lucia) | LN810052 (340 bp) |  |  |
| *Megalomys desmarestii* (Martinique) | LN810053 (459 bp) |  |  |
| *Pennatomys nivalis* (Saint Kitts) | LN810054 (412 bp) |  |  |
| *Pennatomys nivalis* (Nevis) | LN810055 (412 bp) |  |  |

**Table S3.** The estimated mutation rates that would be required when divergence dates between selected oryzomyine taxa are calibrated to the timing of historical events (cyt *b* only data).

| **Taxa** | **Fixed divergence date** | **Mutation rate (% per million years)** | | |
| --- | --- | --- | --- | --- |
|  |  | **Mean** | **95% HPD lower** | **95% HPD upper** |
| St Kitts & Nevis | 7 Kya | 212 | 90 | 348 |
| St Lucia & Martinique | 7 Kya | 100 | 16 | 202 |
| Antigua & Guadeloupe | 7 Kya | 87 | 10 | 193 |
| Antigua, Guadeloupe & *Hylaeamys* | 3.75 Mya | 6.7 | 3.9 | 9.6 |
| St Lucia, Martinique, St Kitts, Nevis & St Eustatius | 3.75 Mya | 5.8 | 3.6 | 8.3 |
| St Lucia, Martinique, St Kitts, Nevis, St Eustatius, *Nesoryzomys* & *Aegialomys* | 3.75 Mya | 7.2 | 4.8 | 10 |
