# Supplementary figures and images for: Unexpected evolutionary diversity in a recently extinct Caribbean mammal radiation
Source: Proc Biol Sci. 2015 May 22;282(1807):20142371. doi: 10.1098/rspb.2014.2371 (PMC4424637; doi:10.1098/rspb.2014.2371)

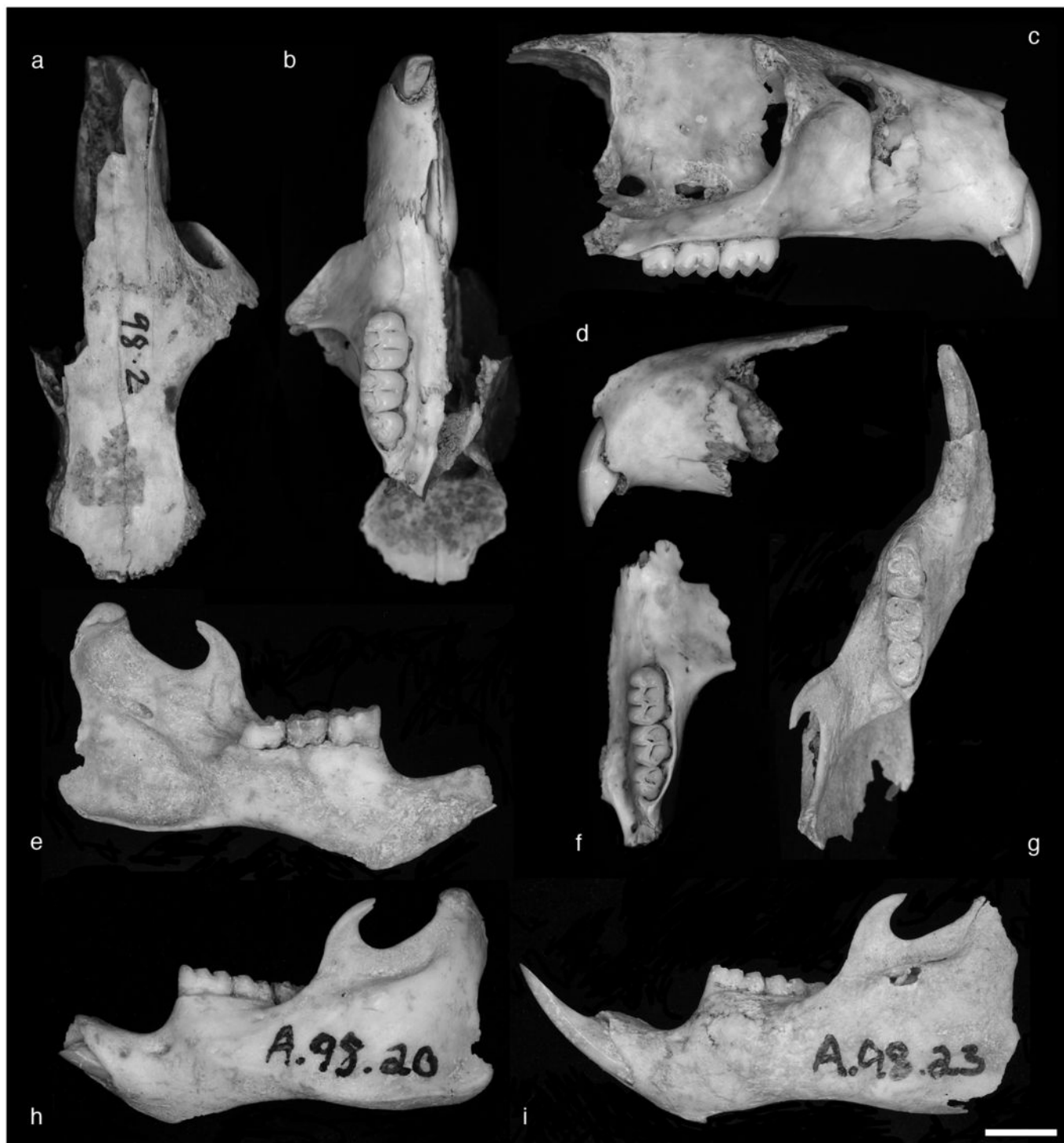

Supplement: Supplementary file 2 [file rspb20142371supp2.pdf]

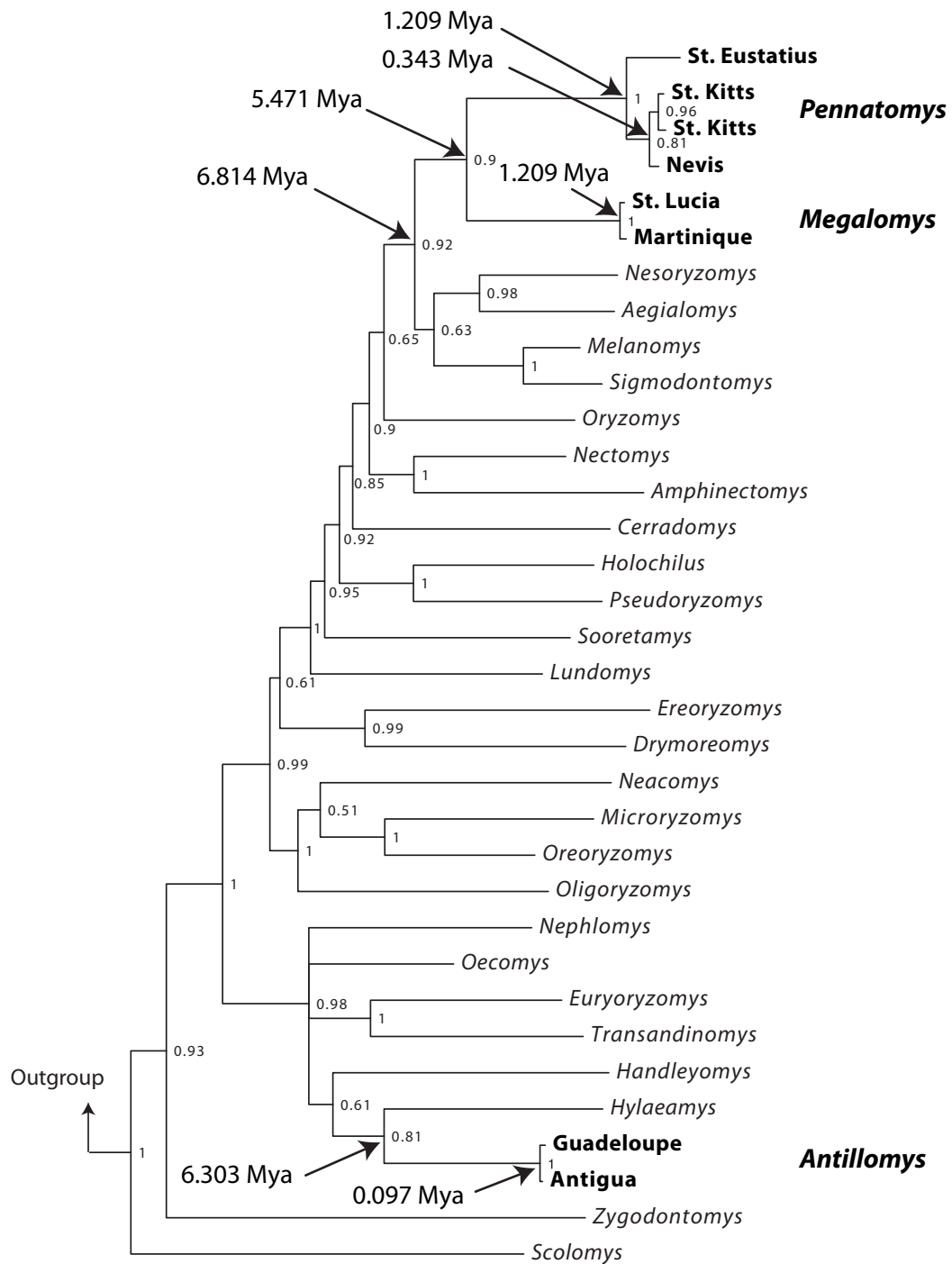

Approximate Geological Timeline Mya

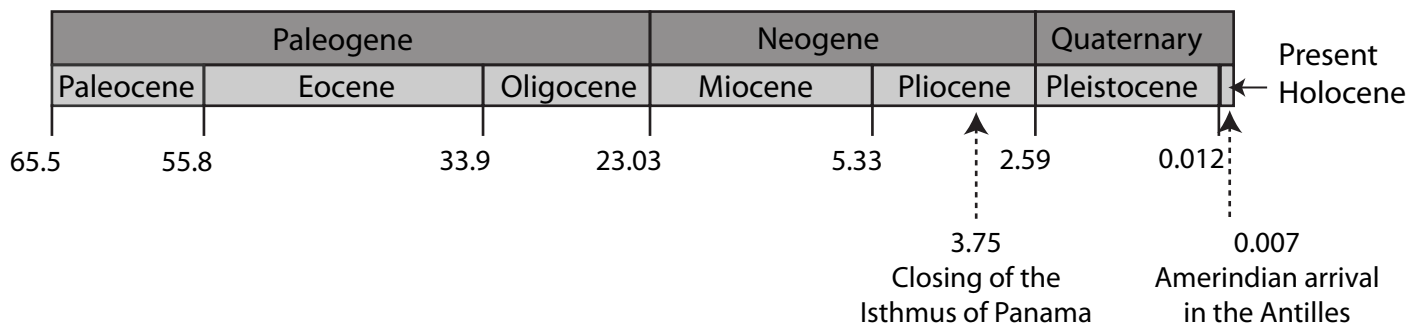

Supplement: SI_Fig_2 [file rspb20142371supp3.pdf]
